# A Simulation of Thermal Management Using a Diamond Substrate with Nanostructures

**DOI:** 10.3390/mi14081559

**Published:** 2023-08-05

**Authors:** Tingting Liu, Kaiwen Zheng, Tao Tao, Wenxiao Hu, Kai Chen, Ting Zhi, Yucong Ye, Zili Xie, Yu Yan, Bin Liu, Rong Zhang

**Affiliations:** 1 Jiangsu Provincial Key Laboratory of Advanced Photonic and Electronic Materials, School of Electronic Science and Engineering, Nanjing University, Nanjing 210093, China; 2 College of Electronic and Optical Engineering & College of Flexible Electronics (Future Technology), Nanjing University of Posts and Telecommunications, Nanjing 210023, China

**Keywords:** diamond, nanostructures, thermal management, comsol, simulation

## Abstract

In recent years, the rapid progress in the field of GaN-based power devices has led to a smaller chip size and increased power usage. However, this has given rise to increasing heat aggregation, which affects the reliability and stability of these devices. To address this issue, diamond substrates with nanostructures were designed and investigated in this paper. The simulation results confirmed the enhanced performance of the device with diamond nanostructures, and the fabrication of a diamond substrate with nanostructures is demonstrated herein. The diamond substrate with square nanopillars 2000 nm in height exhibited optimal heat dissipation performance. Nanostructures can effectively decrease heat accumulation, resulting in a reduction in temperature from 121 °C to 114 °C. Overall, the simulation and experimental results in this work may provide guidelines and help in the development of the advanced thermal management of GaN devices using diamond micro/nanostructured substrates.

## 1. Introduction

In the realm of industrial semiconductor development, silicon (Si) and gallium arsenide (GaAs) have emerged as representative materials for the first and second generations of semiconductors. These materials have attained a high level of technological maturity, accompanied by a wide scope of applications. However, their inherent material limitations pose challenges for silicon-based and gallium arsenide-based semiconductor devices operating under high-temperature, high-frequency, and high-power conditions, thereby impacting their performance. In recent years, gallium nitride (GaN) has emerged as the exemplar of third-generation wide-bandgap semiconductor materials. Over the past ~20 years, numerous investigations have been carried out in the development of gallium nitride materials and their corresponding electronic devices, achieving remarkable breakthroughs. GaN-based semiconductor materials form numerous optoelectronic and power devices with superior performances [1,2]. Recently, GaN-based power devices have demonstrated high breakdown field strength, high two-dimensional electron gas concentration, and high electron saturation rates [3,4]. The output power density of GaN devices can exceed that of Si devices and GaAs devices [5], which make GaN an excellent candidate in advanced power and radiofrequency devices [6,7]. However, there is still much room for GaN materials to improve in the realm of crystal quality. It was not until 1986 that Amano [8] achieved success in producing a high-quality GaN film on a sapphire substrate by introducing a new growth technique using an aluminum nitride buffer layer. This opened up avenues for GaN optoelectronic device applications. Currently, the typical process of GaN production involves the growth of buffer layers on sapphire and Si substrates. While Si substrates offer the advantages of affordability and ample dimensions, they also present significant challenges due to their substantial lattice and thermal mismatch with GaN. During their growth, the epitaxial surface is susceptible to cracking, thereby resulting in a notable abundance of dislocations. Moreover, the utilization of sapphire substrates for growing GaN yields a notably smoother and higher-quality surface. In comparison to conventional silicon-based electronic devices, GaN power devices fabricated on sapphire substrates exhibit superior power density output and energy conversion efficiency. Additionally, sapphire substrate devices enable system miniaturization and lightweight design, effectively reducing the size and weight of electronic devices. This significantly mitigates the expenses associated with the devices’ fabrication and production.

In recent years, there have been significant improvements in GaN power devices, leading to increased device integration and output power, as well as a reduction in device size [9,10,11]. When GaN devices operate at high power levels, they generate a substantial amount of heat. The challenge lies in efficiently dissipating this heat in a timely and effective manner to prevent temperature accumulation within the device. Failure to address this issue will result in an escalating device temperature, commonly referred to as the self-heating effect. Obviously, heat accumulation in high-power devices has become a serious challenge to their reliability and stability [12], mainly due to the low thermal conductivity of GaN and substrates. Rises in temperature also limit the performance of GaN devices under high-power output [13,14,15]. For example, Kuzmik [16] studied the self-heating effect of GaN HEMT devices on sapphire substrates, and found a level of 2.6 W/mm. When localized Joule heating occurs in the 2DEG channel, extremely high temperatures will be generated in the immediate vicinity of the junction hotspot, in the order of a few MW/cm^−2^. Despite its excellent performance compared to other semiconductor materials, its operating temperature can reach 600 °C [17]. However, the issue of the reliability of GaN devices at high temperatures has not been fully settled. As a result, the actual operating temperature of GaN devices might be even higher than simulated temperatures, which greatly limits their high thermal stability. Therefore, the heat management of GaN devices has become one of the main bottlenecks restricting their further development and wider application. Traditional packaging technology is limited by low thermal conductive materials to improve the heat dissipation performance of power devices, as it can only address heat dissipation from the proximal area of the device [18]. However, the incorporation of high-thermal-conductivity materials can greatly enhance the heat dissipation capability of the device [19,20]. In order to improve near-junction heat dissipation ability, diamond, which is the best candidate for use as a thermal conductor, has been used as the substrate for making GaN-on-diamond devices [21,22,23]. Group 4 Labs of Fremont [24], in collaboration with university partners, have developed a process of transferring GaN onto a 2-inch diamond substrate. Lower operating temperatures have been obtained compared to SiC substrates under equivalent conditions. Moreover, the GaN-on-diamond device can be mounted on a larger heat sink, further dissipating the heat away from the device. Liu [25] demonstrated GaN-on-diamond structures with excellent microstructures. Good thermal properties and stability can be achieved by utilizing diamond seed crystals with small particle sizes. In 2021, M.Y. Chernykh [26] introduced a novel approach for the production of highly effective heat sinks tailored to GaN-based transistors. Their method involved the cultivation of polycrystalline diamond coatings on functional Si layers of SOI wafers, followed by the selective etching of thick silicon substrates and thin thermal oxides. Experimental results demonstrated a temperature reduction of over 50 °C compared to conventional GaN-on-SiC HEMT technology.

In this work, a diamond substrate with nanostructures was designed and investigated for GaN-based power devices to improve their thermal management. The thermal distribution of working devices was simulated using COMSOL software under different conditions (conventional sapphire and optimized diamond substrates). The simulation results of the surface optimized diamond nanostructures demonstrated the promising heat dissipation capabilities of diamond substrates, which could support the further development of high-power devices.

## 2. Simulation Experiment

COMSOL Multiphysics software enables all material parameters to be freely defined as a function of arbitrary physical quantities, allowing the study of results arising from the simultaneous action of multiple physical fields. Therefore, in this study, COMSOL Multiphysics (v4.2) software was used for inbuilt modelling and simulation analysis.

**Figure 1:**
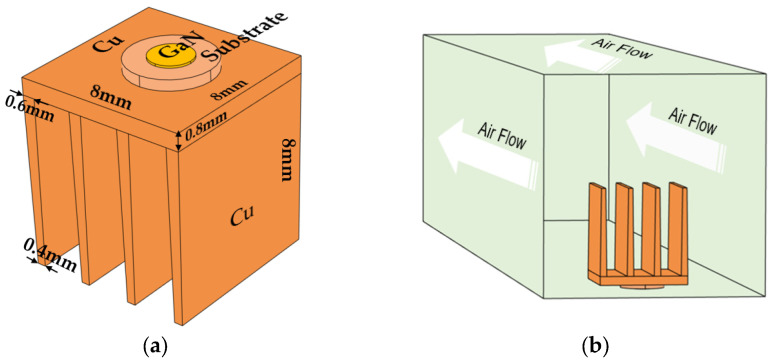
(**a**) Geometric model of GaN device heat dissipation. (**b**) The air fluid flow in the models (white arrows represent the direction of air flow).

A conventional GaN device with sapphire/Si/SiC substrates was proposed as the target device for simulation. We focus on the effect of the substrate on device heat dissipation. The simulation model was constructed to avoid the challenges associated with numerically simulating physical objects, optimizing mesh density while maintaining accuracy. This improved the convergence of the calculation and reduced the calculation time. Therefore, when formulating the model to account for the primary heat source of the entire GaN device, a certain level of simplification in modeling was necessary. The simplified model reduces the complexity while ensuring relatively accurate device temperature results. In this model, the GaN device was attached to a copper heat sink mounted in a channel with a rectangular cross-section. The radiator base is a rectangular shape measuring 8 mm in length, 8 mm in width, and 0.8 mm in height. One side directly contacts the substrate, while the other side is equipped with four fins to aid in heat dissipation. The fins are irregular rectangles, as shown in Figure 1a. During the modeling, we incorporated a domain corresponding to the air channel within the geometry to compute the airflow and temperature field. Air entered the channel through the entrance and escaped from the exit. All other external surfaces were thermally insulated except for the heat transfer surface in contact with the substrate. The direction of air flow in the model is illustrated in Figure 1b. The electronic device was assumed to generate 1 W of heat, which was distributed throughout the device. In this paper, the built-in modeling program in COMSOL was employed to draw the heat dissipation geometric model and conduct the calculation. The simulation primarily involved solid heat transfer and fluid heat transfer modeling to simulate air flow in a natural environment. Two cases were assumed for the contact surface between the substrate and the heat sink, i.e., the ideal no dielectric contact, and the case wherein there was a dielectric interlayer between device and substrate. During the simulation, the GaN device was used as a heat source with a heat generation rate of 1 W, a room temperature of 25 °C, an air flow rate of 0.1 m/s, and a PMMA medium material layer with a thermal conductivity of approximately 0.19 W/(m·K). The specific modeling data and the material coefficients required for the simulation calculation are provided in Table 1 and Table 2.

**Figure 2:**
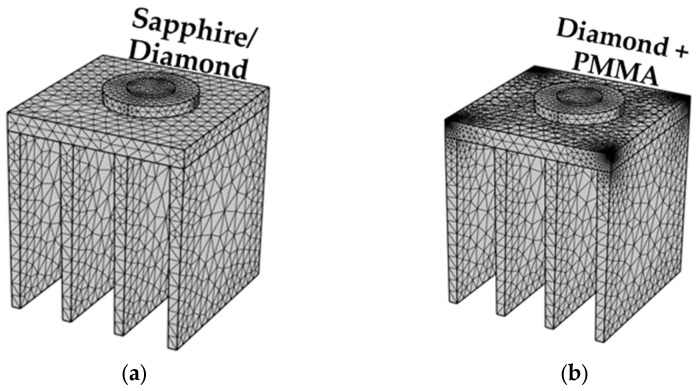

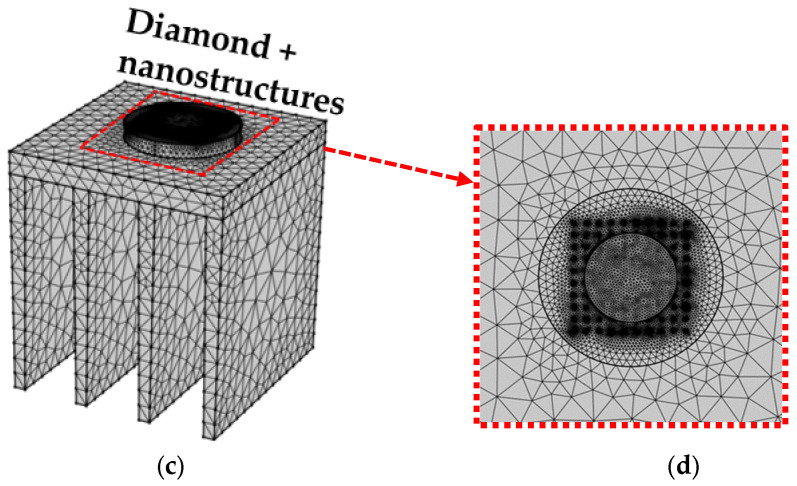
(**a**) Meshing of medium-free modeling. (**b**) Meshing of PMMA dielectric module. (**c**) Meshing of substrate module with nanostructures. (**d**) Enlarged view of the meshing of the micro/nanostructure in Figure (**c**).

According to the geometric model of the module, the simulation was based on the mesh analysis of COMSOL software to mesh the module, and the physical field was used to set the mesh type in the mesh setting. It should be mentioned that the accuracy of the grid was simplified and the fineness of the grid was reduced to improve the convergence of the calculation and to reduce the calculation time. After the meshing, the membrane construction grid without medium contained 88,919 domain cells, 10,629 boundary cells and 927 edge cells. The modeling grid in the presence of PMMA medium contained 185,133 domain cells, 17,053 boundary cells and 1393 edge cells. The modeling group of the substrate with micro- and nanostructures contained a finer grid with a larger number of meshes. The meshing structures of all the modeling samples are shown in Figure 2.

## 3. Results and Discussion

### 3.1. Analysis of Heat Dissipation from Different Substrates

**Figure 3:**
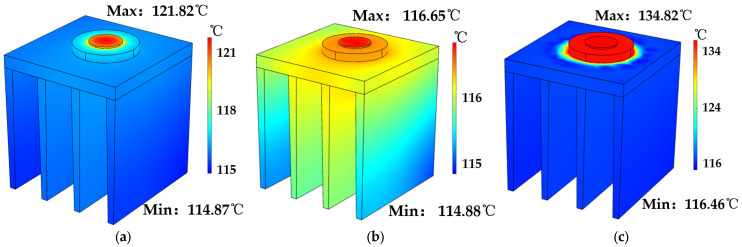
(**a**) Sapphire substrate without dielectric. (**b**) Diamond substrate without dielectric. (**c**) Diamond substrates with PMMA interlayer.

In order to reveal the heat dissipation in different contact modes, the simulation considers both the ideal contact and the presence of a dielectric layer, where the dielectric layer is PMMA. In this work, we assume the GaN devices were grown on sapphire and diamond substrates, followed by the integration of the complete device onto a copper heat sink. Figure 3 displays the temperature distributions of the two substrates in the ideal and non-ideal contact conditions. Specifically, Figure 3a shows the sapphire substrate in the ideal case, Figure 3b shows the diamond substrate in ideal case, and Figure 3c shows the diamond substrate under heat dissipation with the presence of the dielectric layer.

Figure 3 demonstrates significantly enhanced heat dissipation in diamond substrates compared to those of sapphire substrates. Under ideal conditions, the maximum temperature of the GaN chip on conventional sapphire substrate reaches approximately 121 °C, while that on diamond substrate is 116 °C. The superior thermal performance of the diamond substrate can be attributed to its significantly higher thermal conductivity compared to that of sapphire [10,28]. Even with a PMMA dielectric inter-layer, the maximum temperature of GaN chip on diamond only rises to 134 °C. Based on these simulation results, it can be found that the diamond substrate with high thermal conductivity can make the most contribution in heat dissipation. It should be mentioned that the thermal dissipation tests could be influenced by hetero-interface thermal resistance. In addition, the micro/nanostructures on the diamond surface need to be designed and optimized, which might further enhance its heat dissipation performance.

### 3.2. Analysis of Heat Dissipation on Different Surfaces of Diamond Substrates

To further enhance the heat dissipation, the design of three typical micro/nanostructures was proposed and simulated, which include cylindrical, square, and hemispherical shapes. Different parameters for micro/nano columns were simulated using COMSOL software to find out the optimized structures for heat sink.

**Figure 4:**
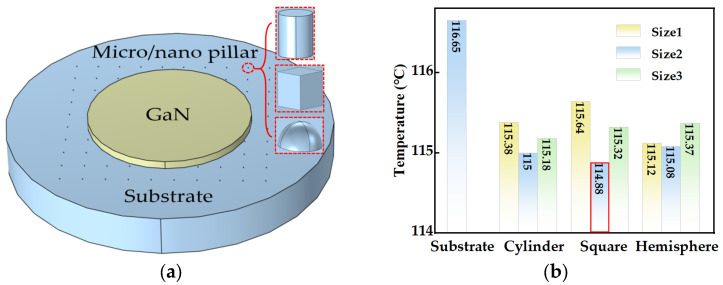
(**a**) Schematic diagram of the substrate micro/nanostructure (The red boxes indicate the three types of micro/nano-columns on the surface of the patterned substrate). (**b**) Effect of different micro/nanostructures on the surface of diamond substrates on heat dissipation.

The shape of the micro/nanostructures served as the sole variable in the modeling process. Therefore, it is imperative to maintain consistent dimensions of the various types of nanopillars. As shown in Table 3, each group was established based on the surface area of the cylindrical micro/nanostructure, and the spherical radius and square column side heights were adjusted to maintain the same interface area. The maximum temperatures for all samples are presented in Table 3. Additionally, Figure 4a illustrates the structure and simulation results of the group of substrates with different-sized micro/nanostructures.

Figure 4 illustrates that the device temperatures can be further reduced when a substrate with a micro/nanostructure is employed. This means that surface-structured substrates will enhance device heat dissipation. In this paper, it is important to mention that simulation calculations were conducted to compare different shapes of micro/nanostructures while maintaining a consistent contact interface area. The results in Table 3 reveal that the hemispherical micro/nanostructured devices provide a better thermal performance, with a low temperature of 115.12 °C. Among the first set of dimensions, the thermal performance decreases in the following order: hemispherical, cylinder and square. Of course, it can be expected that the sizes for each micro/nanostructure will also have influence on heat dissipation. As the size of the micro/nanostructure increases, the device temperature will decrease. This implies that an increased contact interface area between the substrate and device may contribute to enhanced heat dissipation. However, it is noteworthy that once the size of the nanopillar is over a certain value, the temperature begins to increase instead. In this work, an investigation was carried out by distributing an equal number of nanopillars across areas of varying sizes. These nanopillars have a uniform shape and size throughout the study. The obtained results indicate a relationship between the size and the maximum temperature of the device. The sample with smaller area has higher maximum temperatures. This suggests that a denser distribution of the same number of nanopillars corresponds to a deteriorated thermal performance of the substrate. It is noteworthy that among the simulated size groups, the substrate featuring cylindrical micro/nanostructures demonstrates optimal heat dissipation performance. Within the simulated groups, the device temperature reaches its lowest point at 114.88 °C.

**Figure 5:**
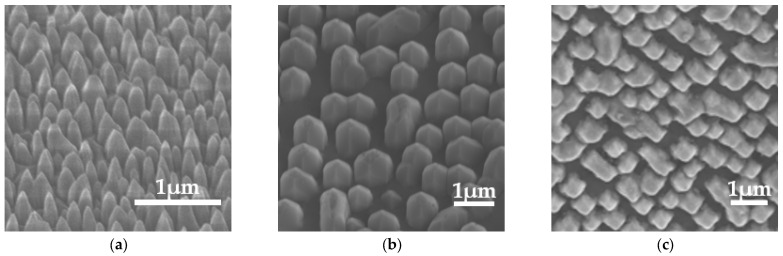
(**a**) Cylindrical micro/nanostructure substrate. (**b**) Hemisphere micro/nanostructure substrate. (**c**) Square column micro/nanostructure substrate.

It should be explained that the micro/nanostructures were set as the ideal conditions for simulation, while the acquired diamond samples were subjected to the limits of fabrication methods. Firstly, the growth of a single-crystal diamond substrate was carried out using microwave plasma chemical vapor deposition (MPCVD, Opto-Systems ARDIS-300). Prior to initiating the growth of monocrystalline diamond, meticulous pretreatment of the seed crystals was conducted to eliminate surface impurities. The pretreatment process involved a H_2_ plasma etching at 900 °C for 30 min with a pressure of 250 Torr and an input microwave power of 3000 W. During the process of epitaxial growth of the diamond, it was common for the edge temperature to exceed that of the central surface temperature. Consequently, this phenomenon promoted polycrystalline nucleation along the edges of diamond substrates, thereby reducing the available area on the single crystal surface. To effectively suppress the occurrence of polycrystalline growth at the edges, a strategic approach involved the incorporation of a circular Mo bracket within the central region of the CVD reaction chamber [29]. By implementing this technique, the seed crystals can be accurately positioned, ensuring optimal crystallization quality. Next, a self-organized nickel mask using rapid thermal treatment and a top-down plasma etching technique was adopted to fabricate nano-dwellings. This process offers cheap way of acquiring nanopillars, ranging from tens of nanometers to hundreds of micrometers. Other methodologies such as nano-imprinting, electron-beam lithography, and deep UV lithography can also be adopted to provide a highly ordered micro/nano pattern. Subsequently, the patterned diamond underwent etching using a combined ICP (inductively coupled plasma, Oxford-ICP100) and MPCVD etching process. As is illustrated in Figure 5a, a cylindrical micro/nano diamond nanopillar structure can be acquired via ICP etching with conditions including RF/ICP power: 100/800 W, gas flow O_2_: 20 sccm, and pressure: 200 mtorr. The height of the resulting nanopillars is determined by the duration of the etching process. After the ICP etching process, hydrogen plasma etching was conducted to form a diamond nanostructure close to a hemispherical shape, as shown in Figure 5b. The hydrogen plasma was carefully controlled with the conditions including microwave power: 2000 W, gas flow H_2_: 100 sccm, and pressure: 150 torr. In order to obtain a diamond nanostructure similar to the square design in Figure 5c, the diamond substrate was etched with hydrogen plasma, with only a Ni mask. Afterwards, all diamond substrates with nanostructures were subjected to cleaning in a 1:1 dilute nitric acid solution. This cleaning procedure aimed to eliminate any residual Ni particles present in the seed crystal. The nanostructured diamond substrate, which was prepared as described above, underwent observation utilizing scanning electron microscopy (SEM), as depicted in Figure 5. Frankly, the high hardness of the diamond material makes it hard to acquire well-organized cylindrical, hemispherical and square column micro/nanostructure designs. The substrate samples prepared in the experiment did not meet the expected standards, as the shapes of the nanopillars lacked standardization, and the dimensions were not sufficiently precise.

With the continuous advancements in semiconductor technology, conventional semiconductor materials are no longer sufficient to meet the demands of high-temperature and high-frequency applications in terms of material properties and device functionalities. In the near future, GaN-based power devices will be integrated together with other units to form highly compacted IC chips, wherein thermal management will be a big issue to be settled. However, diamond-related heteroepitaxial growth is currently a hot and difficult topic, wherein the lattice and thermal mismatch are big issue. Hetero-integrated devices will encounter challenges due to the significant coefficient of thermal expansion disparity between diamond and GaN, resulting in a tendency to detach [30,31,32]. Thus, it can be expected that diamond-related heteroepitaxial growth and hetero-integration will be the next hot research topics, which are necessary to enhance device performance. It is important to acknowledge that there are still certain limitations in the present research, thereby emphasizing the need for further investigations in future studies.

1. The fabrication of diamond nanostructures was not well achieved, and the micro/nanostructures on the diamond surface did not create the desired shape due to the limitations imposed by the existing processes and equipment. In the future, the utilization of nanoimprinting technology will give rise to uniform columns. Efforts will be made in etching methods to enhance the nanopillar preparation process, enabling the fabrication of finer nanostructures on the diamond surface.
2. The integration of GaN and diamond will be investigated, wherein significant disparity in thermal expansion coefficients between GaN and diamond need to be settled. The growth of GaN on a single-crystal diamond substrate should be carried out. Further refinement is necessary to enhance the epitaxial growth process of GaN on diamond.
3. Experimental methods for the thermal testing of diamond-based GaN devices will be studied for the sake of achieving enhanced thermal management. Further endeavors and research are imperative to address this issue and explore potential solutions.

## 4. Conclusions

In this work, a diamond material with high thermal conductivity was employed as a substrate to improve device heat dissipation. We endeavored to optimize the substrates by introducing micro/nano columns of various sizes and structures. The results can be summarized as follows. (1) The most effective heat dissipation structure optimized in this investigation features square nanopillars with a height of 2000 nm. The nano-square diamond column substrate led to a reduced temperature as low as 114.88 °C. (2) Experimentally, single-crystal diamond substrates with nanostructures were prepared via CVD and a top-down etching process, and these were close to the nanostructures in their design. In the future works, GaN devices will be transferred onto diamond substrates with micro/nanostructures.

The results in this paper are helpful for addressing the issue of chip-level heat accumulation and further enhancing the power output potential of GaN devices, thus providing insights into the development of GaN power devices with high heat dissipation capabilities. Of course, more efforts are needed. Additionally, we believe high-power devices can be freed from the constraints of thermal management in the near future.

## Figures and Tables

**Table 1:** Modeling size.

Modules		Data
Radiator	x-directional base dimension	8 mm
	y-directional base dimension	8 mm
	z-directional base dimension	0.8 mm
	x-direction fin size (top)	0.4 mm
	x-direction fin size (bottom)	0.6 mm
	Height of fins	8 mm
	Number of fins	4
Substrate	R	2000 μm
	H	500 μm
Device	R	1000 μm
	H	100 μm

**Table 2:** Material coefficient.

Material	Heat Conductivity [W/(m·K)]	Constant-Pressure Heat Capacity [J/(kg·K)]	Density [g/cm^3^]
Sapphire	25.12 (@100 °C) [27]	-	3.98
Diamond	2000	516	3515
GaN	130	490	6070
PMMA	0.192	1420	1190
Cu	400	385	8960

Other data are sourced from COMSOL software.

**Table 3:** Parameters and results of micro and nanostructures for heat dissipation.

Substrate Construction	Size 1 (nm)	Size 2 (nm)	Size 3 (nm)	T 1 (°C)	T 2 (°C)	T 3 (°C)
Square column	L: 561	L: 2097	L: 5985	115.64	114.88	115.32
Cylinder	R: 200	R: 1000	R: 3000	115.38	115.00	115.18
	H: 1150	H: 3000	H: 8000			
Hemisphere	r: 500	r: 1871	r: 5359	115.12	115.08	115.37

## Data Availability

All relevant data are available from the corresponding author upon reasonable request.
